# Innovations, recipients, and contexts in the implementation of pediatric neurocritical care programs: a multisite qualitative study refining i-PARIHS

**DOI:** 10.64898/2026.10.13.26365431

**Published:** 2026-10-14

**Authors:** Mia T. Vogel, Jessica S. Wallisch, Bradley J. De Souza, Pradip P. Chaudhari, Asmaa Hassan, Anvi Jampani, Katherine M. Steffen, Jose A. Pineda

## Abstract

**Background:**

Pediatric neurocritical care (PNCC) has emerged as a distinct field, and many hospitals have moved to establishing dedicated PNCC programs. How practice change is implemented within these programs is not well understood. This study had two objectives. First, we sought to understand the barriers and facilitators of practice change in PNCC from the perspectives of the multiprofessional clinicians who deliver it. Second, we sought to characterize how well the integrated Promoting Action on Research Implementation in Health Services (i-PARIHS) implementation determinant framework captures them.

**Methods:**

We conducted semi-structured interviews with a purposive sample of clinicians involved in PNCC at three U.S. academic medical centers. The interview guide was developed using i-PARIHS to organize barriers and facilitators of practice change across the innovation, recipients, context, and facilitation constructs. We applied framework analysis, combining deductive i-PARIHS, study-specific, and inductively derived categories. Two coders independently indexed the transcripts and reconciled them to consensus, with first-author adjudication. Preliminary site-level findings were validated with participants.

**Results:**

We interviewed 33 clinicians: 24 physicians, 7 nurses, and 2 advanced practice providers. Most determinants of practice change mapped to existing i-PARIHS constructs, but three were not adequately captured by the framework as currently specified. The most consequential was an interprofessional authority gradient that determined whose preferences prevailed when intensive care, neurology, and neurosurgery disagreed. The other two were clinician autonomy, which served as the lens through which the acceptability of a change was judged, and the affective state clinicians bring to practice change. Receptivity depended more on recipients’ values, prior experience, and capacity to absorb change into a demanding workflow. Facilitation was variable, frequently under-resourced, and dependent on credibility across professional groups. We translated these findings into three propositions linked to implementation strategies and outcomes.

**Conclusions:**

Participants attributed practice change in PNCC more to the recipients of change, the multiprofessional context, and the availability of facilitation than to the strength of evidence for a given practice. i-PARIHS captured most barriers and facilitators well but may be adapted in this setting. Credible, resourced, authority-sensitive facilitation may improve the prospects for sustained practice change as institutions develop PNCC programs.

**Contributions to the literature:**

- Determinant frameworks are widely used but rarely revised once published; this study supports three i-PARIHS framework modifications potentially applicable to the general pediatric ICU and PNCC programs regarding clinician autonomy, the affective state clinicians bring to change, and the interprofessional authority gradient.
- We identify authority-sensitive facilitation as an important driver of sustained practice change amongst multispecialty teams.
- We offer three i-PARIHS elaborations with linked implementation outcomes and implications for selecting implementation strategies for teams with shared authority across professions.

